# Components and Related Factors of Nursing Interventions for Improving Resilience in Cancer Patients Undergoing Chemotherapy

**DOI:** 10.3390/healthcare9030300

**Published:** 2021-03-08

**Authors:** Shiena Shimada, Michiko Aoyanagi, Naomi Sumi

**Affiliations:** 1 Department of Nursing, Hokkaido University Hospital, Sapporo 060-8648, Japan; romio.shiena0923@gmail.com; 2 Faculty of Health Sciences, Hokkaido University, Sapporo 060-0812, Japan; aoyanagi@med.hokudai.ac.jp

**Keywords:** resilience, cancer, nursing, chemotherapy

## Abstract

Resilience is considered an effective concept for cancer patients, but nursing interventions for improving resilience have not been studied adequately yet. We aimed to explore the components and related factors of nursing interventions for promoting resilience among cancer patients receiving chemotherapy (NIPRPC). This cross-sectional study included 68 facilities from 396 cancer hospitals in Japan. Participants were 377 nurses who worked at the outpatient chemotherapy center or cancer ward. They completed self-administered questionnaires including the NIPRPC items and Resilience Scale for Nurses, etc. We conducted factor, correlational, and regression analysis. Based on the exploratory factor analysis, six-dimensional factor components: “Support for patients during the present situation and increasing their self-affirmation”, “Support for self-help”, “Support for utilizing the cancer medical team”, “Support for obtaining family cooperation”, “Support for maintaining regular lifestyle during chemotherapy”, and “Support for interacting with cancer patients and utilizing necessary information”. The strong related factors for all six factors were the resilience of the nurses and the number of collaborations with multiple occupations. Our findings will help nurses improve the psychosocial quality of life of cancer patients and address their needs related to cancer chemotherapy treatment.

## 1. Introduction

Cancer patients undergoing chemotherapy experience various physical and psychological symptoms [1,2]. Chemotherapy is associated with distressing symptoms, impaired quality of life, and psychological distress. Resilience is the ability to cope with adversity and to return to a healthy psychological state. Although there is a slightly different definition of resilience in a previous study, most of the proposed definitions include a concept of healthy, adaptive, or integrated positive functioning during a period after experiencing adversity [3]. According to Molina’s review, resilience among patients with cancer has been characterized in three ways in existing literature throughout the cancer continuum (i.e., as a baseline characteristic, as a mechanism to promote positive outcomes, and as an outcome itself) [4]. Cancer patients often repeatedly experience adversity associated with their health condition and treatment. The concept of resilience in research and practice in cancer care and also unique insights on factors and dynamic process facilitating adult survivors to face the significant adversity associated with cancer are promising [5]. In previous research, resilience is effective for reducing psychological distress [6,7], depression [7,8], and fatigue [9] in patients. Moreover, resilience promotes well-being, such as the QOL [9,10,11,12] and satisfaction with life [13,14]. Furthermore, it is reported that resilience is positively associated with QOL and may comprise a robust buffer between depression and QOL in chemotherapy patients [15].

Several interventions for resilience in cancer patients have been developed in previous studies [16,17,18,19,20]. These interventions were useful for improving resilience but focused on the cognitive or psychological aspects. Apart from these, we wondered if there was an effective way to increase resilience in holistic nursing practice. According to this point, a previous study stated that nurses have practiced patients support so that patients can accept the unexpected reality arising from health problems and be able to overcome the crisis caused by the situation [21]. Furthermore, previous studies stated that current nursing care practices included resilience promotion even without any new intervention [22]. In addition, since it has been reported that the nurses’ emotional well-being supports their work as well as the effect it has on patient care [23], we expected nurse’s own resilience correlates to offering nursing interventions for promoting resilience in cancer patients. This study had two objectives based on the above: to explore (1) the components of nursing interventions for promoting resilience in cancer patients receiving chemotherapy and (2) related factors affecting these components of nursing interventions. Resilience in this study was defined as a patient’s ability to restore and maintain a stable psychological state and adapt to adversity and temporary psychological depression.

## 2. Materials and Methods

This was a descriptive quantitative cross-sectional study. Out of 396 cancer hospitals in Japan’s 47 prefectures, we requested 152 cancer hospitals in 10 prefectures to participate in this study in September 2016. Prefectures were selected based on population ratios and area classification. Questionnaires were distributed in October to 68 facilities that agreed to participate in the survey. We asked the nursing department staffs to select nurses as appropriate subjects for this study and distribute questionnaires to each nurse. We asked them to recruit 10 nurses (i.e., 5 nurses working for the outpatient chemotherapy center and 5 nurses working for the cancer ward, which mainly offers chemotherapy to gastrointestinal cancer patients). The inclusion criteria were the following. Certified nurse specialists in cancer nursing (CNS) or certified nurses (CN) were included in this study. Nurses incharge were excluded from this study because nursing incharge does not provide direct care to patients. We conducted a factor analysis to clarify the structuring of nursing practices that promote resilience in cancer patients. In the factor analysis, it is considered that five people are required for one item [24]. Thus, if five people are required for 65 items, 325 people are needed for this study. The response rate was set to 50%, and the sample size was set to 650 people. Finally, we distributed 678 questionnaires to 68 facilities. The nurses were asked to return the completed questionnaires via mail to the researcher at Hokkaido University. Data was collected between September and October 2016.

### 2.1. Measurement Tool

We used two measurement. One was the survey questions of nursing intervention for promoting resilience in cancer patients receiving chemotherapy (NIPRPC). We specifically made items based on previous reports on resilience in patients or nursing interventions based on resilience as well as our previous study where we interviewed 1 CNS in cancer nursing, 4 CNS, and 2 nurses working at chemotherapy centers based on the advice of CNS in cancer nursing [25,26,27,28,29,30,31,32]. We used the data from the interview and increased its abstraction level by analyzing it by qualitatively and inductively. Based on the above, we tried to ensure content validity. This list includes 65 items with a 4-point Likert scale wherein 1: “Not at all”, 2: “Not so much”, 3: “Sometimes”, and 4: “Always”. In addition, we refined the contents of the question items while being supervised by two researchers with cancer nursing experience.

The second instrument was the Japanese version of the Resilience Scale for Nurses (RSN) that measures the magnitude of nurses’ adaptability to adverse circumstances [33]. This tool has been used in many studies on nurses’ resilience in Japan [34,35,36,37]. The RSN comprises 4 subscales: “Positivity in nursing”, “Interpersonal skill”, “Having an anchor in personal life”, and “Response to novelty”. Twenty-two items are rated on a 5-point Likert scale ranging from 1 (“No”) to 5 (“Yes”). Possible total scores range from 22 to 110, with higher scores reflecting higher resilience. Pretest was done to check to the validity of questionnaire.

Apart from these two measurement tools, we included items regarding nurses’ personal details in the questionnaire such as age, gender, years of nursing experience, job title, highest education, qualification of cancer nursing specialization, certification in nursing, and the number of collaborations with professionals (palliative care team, psychiatry liaison, etc.)

### 2.2. Statistical Analysis

Descriptive statistics were calculated. The NIPRPC was analyzed with exploratory factor analysis using the maximum likelihood method to investigate factor components of NIPRPC. We selected the promax method because we presumed a positive correlation between each factor and explored component. We calculated the factor scores that indicates the implementation of the NIPRPC. To examine the differences in the NIPRPC list scores by personal data, we conducted a *t*-test and one-way ANOVA. The Tukey test was used for multiple comparisons. The correlation between the RSN and NIPRPC list scores was examined using Pearson’s correlation coefficient. Once the variables associated with NIPRPC list were identified by correlational analyses, they were entered into stepwise regression analysis to determine the variables that significantly predicted the implementation of nursing intervention. Categorical variables were recorded as dummy variables. Multicollinearity was tested, and no variables showed a variance inflation factor greater than 10. The normal distribution of the residues was tested using a normal probability plot for residues.

All analyses were performed using the IBM SPSS Statistics for Windows software version 22.0 (IBM Corp., Armonk, NY, USA).

### 2.3. Ethical Approval

Approval of this study was obtained from the A University Ethical Review Committee, Japan. Participants were informed that participation was completely anonymous and voluntary by documents. We promised that the collected data will only be used in this study. Participants were informed that this survey’s data is confidential, and questionnaires were returned directly to the researchers.

## 3. Results

### 3.1. Participant Characteristics

We distributed 678 questionnaires to 68 facilities and collected 403 questionnaires (the collection rate, 59.4%). We excluded 26 questionnaires due to incomplete responses. Finally, 377 responses were considered valid for the analysis (effective response rate: 93.5%). The demographic characteristics of the participants are shown in Table 1. This study included 365 (96.8%) women and 10 (2.7%) men. Most participants were in their 30s (*n* = 146, 38.7%) and 40s (*n* = 128, 34.0%).

### 3.2. Factor Analysis Findings

Based on descriptive statistic, 8 items appeared to have a ceiling affect, but we considered these items important in the NIPRPC list. Therefore, we did not exclude them and conducted the factor analysis with all 65 items. No items were shown to have a floor effect or Spearman’s rank correlation coefficient of ≥0.8. After performing exploratory factor analysis (maximum likelihood method, promax rotation) of the 65 items in the NIPRPC list, 6 factors, including 27 items, were extracted as Table 2 shows. In the process of exploratory factor analysis, we repeated analysis according to following adoption standard we made: (i) Initial eigenvalues is over 1.0, (ii) Communality after factor extraction is over 0.3, and (iii) Factor loading is over 0.4 for one factor and less than 0.3 for other factors. Based on a literature review, we set the factor loading cut-off to 0.4 [24]. The cumulative contribution rate was 50.14%. Kaiser–Meyer–Olkin measure of sampling adequacy was 0.912. Bartlett’s test of sphericity was significant (χ^2^ = 4339.33, *p* < 0.001). The Cronbach α coefficients were as follows: overall, 0.92; first factor, 0.83; second factor, 0.84; third factor, 0.78; fourth factor, 0.82; fifth factor, 0.81; and sixth factor, 0.67.

### 3.3. Components of Nursing Interventions for Promoting Resilience in Cancer Patients Receiving Chemotherapy

The instrument’s factors were named according to the underlying construct related to the items. The factors were labeled as “Support for patients during the present situation and increasing their self-affirmation” (factor 1), “Support for self-help” (factor 2), “Support for utilizing the cancer medical team” (factor 3), “Support for obtaining family cooperation” (factor 4), “Support for maintaining regular lifestyle during chemotherapy” (Factor 5), “Support for interacting with cancer patients and utilizing necessary information” (factor 6). Individual factor scores were as follows: factor 1 was 23.3 ± 2.8 (Rating score: 83.2%); factor 2 was 14.4 ± 2.7 (72.0%); factor 3 was 16.2 ± 2.4 (81.0%); factor 4 was 8.5 ± 1.8 (70.8%); factor 5 was 9.0 ± 1.8 (75.0%); and factor 6 was 11.0 ± 2.1 (68.8%).

### 3.4. Factors Related to Nursing Interventions for Promoting Resilience in Cancer Patients Receiving Chemotherapy

Table 3 and Table 4 summarize the relationship between the six factors of the NIPRPC list, the nurses’ individual factors (position, professional qualifications, learning resources related to nursing practice, and resilience score), and the number of collaborations with professionals. Although there was no fixed trend in group means based on age or years of experience, nurses with qualification had significantly higher scores than nurses without qualification (*p* < 0.05). Nurses with job titles scored higher in the 3rd and 5th factors (*p* < 0.05). Nurses studying at academic societies had higher scores for factors 2, 5, and 6 (*p* < 0.01). The average number of collaborations with professionals was 8.1 ± 2.5.

Nurse resilience was found to correlate with all six factors (r = 0.188~0.325, *p* < 0.01). Similarly, the number of collaborations with professionals was found to be correlated with all six factors (r = 0.170~0.213, *p* < 0.01). Following these analyses, we conducted a multiple regression analysis based on the results of the group comparison. Table 5 presents the results of the multiple regression analysis for six factors of NIPRPC items. Two important related factors for all six factors are the number of collaborations with professional (β = 0.124~0.173, *p* < 0.01) associated with “nurse resilience” (β = 0.123~0.300, *p* < 0.000). For other factors and important indicators, the second and sixth factors were professional qualifications (β = 0.160~0.141, *p* < 0.05), the third factor was nurse with job titles (β = −0.106, *p* = 0.05), and the fifth and sixth factors showed learning activity in academia (β = 0.142, *p* = 0.01).

## 4. Discussion

### 4.1. Components of the Nursing Interventions

NIPRPC included elements like self-affirmation, coping, utilization of professionals other than nurses, family, personality, interchange and information exchange among cancer patients, etc. According to the first factor, previous study investigated construction of cancer patients’ resilience and stated that positive change is promoted by being conscious of self-acceptance when you improve positive sense [38]. Besides personality, sense of values and positive recognition for external environment including people are considered as factor to maintain resilience of breast cancer patients who experienced treatment [29]. Coping is considered as an attribute of resilience [38,39]. It is reported that you can live life that it seems to be oneself as a factor to promote resilience of breast cancer patients [40]. They are each in agreement with second factor or fifth factor. In third, fourth, and sixth factors, contents related to social support were extracted. Social support is considered an important element in resilience concept [4,39,41,42]. The third factor is support for a patient to get support and information from the medical staff except nurse, and it may be said that it is to enlarge the social support of the patient. The fourth factor had contents to maintain the support relations of the patient-family well and confirm satisfaction for the support from family. Social support satisfaction is high, and resilience tends to be high [43]. Therefore, it is thought to promote resilience if nurses have patients build a good relationship with a supporter or get support in line with the needs of the patient. The sixth factor showed social support from cancer patient. Zeng’s program to improve resilience utilizes the same disease patient [20]. In addition, about the effectiveness of the relation between same disease patients, it is suggested that interchange with the same disease patient brings mental stability and forward thinking about the future [44]. According to concept analysis, resilience is defined as the ability to recover from perceived adverse or changing situations, through a dynamic process of adaptation, influenced by personal characteristics, family and social resources, and manifested by positive coping, control and integration [45]. NIPRPC includes those kinds of elements. Overall, it can be said that the six factors have reasonable contents for increasing resilience in cancer patients receiving chemotherapy because they reflect the concept of resilience and factors important for stress coping or psychological health of cancer patients. Our results included “maintain regular lifestyle” content, but it is an uncommon element in other research fields. This may suggest that it is characteristic of nurses, as a viewpoint of interventions, to promote resilience in cancer patients. We investigated contents of the nursing intervention to promote resilience in cancer patients receiving chemotherapy and finally obtained a result to suggest the support with not only cognitive and psychological viewpoints but also holistic. We thought this is a new suggestion in that NIPRCP contained viewpoints of nurse capturing important elements related to patients in cancer nursing.

### 4.2. Related Factors for Nursing Interventions for Promoting Resilience in Cancer Patients Receiving Chemotherapy

We found that “nurse’s own resilience”, “qualification (CNS, CN)”, “job title”, “Use of nursing journals/research papers/academic conferences for learning”, “collaboration with professionals (palliative care team, psychiatry liaison team and pharmacist, etc.)”, and “studying at academic societies” were related to practicing NIPRPC.

A CNS has deep knowledge and skills in a specialized nursing field and efficiently provides nursing care with high standards to individuals, families, and groups having complicated and difficult-to-solve nursing problems. A CN is capable of performing a high level of nursing practice using expert nursing skills and knowledge in a specific nursing field. These qualifications are obtained through an educational curriculum established by the Japan Nursing Association. Therefore, CNS and CN who receive professional education perceive the characteristics and needs of cancer patients sensitively and are usually able to support patients by focusing on their psychosocial problems and strengths than nurses without qualifications. It is also considered nurses with job title have extensive clinical experience. They similarly know the characteristics and psychosocial needs of cancer patients form many cases. In addition, even with regard to nurses learning from nursing journals/research papers/academic conferences, they have advanced knowledge about cancer nursing based on the latest patient needs. That is why it is suggested that nurses like above can provide nursing to promote patient’s resilience.

Regarding the effect of palliative care team on cancer patients, it has been reported that the improvement in edema, fatigue, dry mouth, abdominal distention, and spiritual well-being was significant in the group that received palliative care [46]. The liaison team also contributes to the improvement in the quality of medical care [47]. Thus, nurse’s cooperation by introducing palliative care and liaison teams, and encouraging patients to use them can help patients cope with problems or get psychosocial support. We can infer that nurses who understand the roles and effects of these psychosocial experts and collaborations with them recognize the importance of support so that patients can deal with psychosocial problems, and they also provide NIPRPC. Nurses’ resilience significantly and positively correlated with the NIPRPC score. From the multiple regression analysis, the strongest related factor of NIPRPC was found to be nurses’ resilience. Few studies have examined the relationship between nurses’ resilience and patient resilience. In future studies, we will survey how nurses’ resilience affects the resilience of cancer patients from every aspect, including psychological, physical, and cognitive. So far, the studies on nurses’ resilience have been done from the aspect of occupational hygiene. It has been suggested that nurses’ resilience is effective on nurse’s own work continuation and mental health [48] and that resilience mediates between burnout and psychological distress of nurses [49]. This study suggests the possibility of resilience being effective for nursing practice. Researchers reported that the nurses’ emotional well-being supports their work as well as the effect it has on patient care [43]. Highly resilient nurses exhibit significant correlations to high-level professional activities, positive coping strategies, and awareness of personal and professional boundaries [50]. It suggests that active cooperation with professionals (palliative care team, psychiatry liaison, etc.) may promote patient resilience. It can be said that our results support this view. It may be beneficial for future studies to pay attention to the effectiveness of nurses’ resilience on patient care. From the above, we suggest that the promotion of NIPRPC could be influenced by the in-hospital education to raise nurse’s resilience, the improvement of cancer nursing education by CNS or CN, and the cooperation system with other professionals.

### 4.3. Limitations

There are some limitations in this study. Although we could provide information related to the content of the nursing interventions for promoting resilience in cancer patients receiving chemotherapy, we did not examine whether these interventions could affect patient resilience. Therefore, it is necessary to verify nursing practices that promote patients’ resilience through intervention studies in future research. Besides, psychosocial support needs seem to be unmet among cancer patients [51]. It is also necessary for future studies to check whether these interventions are needed by the patients and examine resilience intervention by nurses using a survey targeting patients. Moreover, since this study targeted cancer hospitals, it is possible that there were many nurses who were able to provide support. Social desirability bias may be another major factor that may be responsible for the high NIPRPC score. In the future, it is important to investigate by broadening the scope of the target hospital towards equalization of cancer nursing. Additionally, the future study, including other independent variables, will deepen knowledge of the relationship between nurses’ own resilience and the implementation of NIPRPC. Thus, it is considered that examining this will be the next issue.

## 5. Conclusions

In this study, the nursing interventions for promoting resilience in cancer patients receiving chemotherapy consist of 6 components, including 27 items related to nursing care. Further, we explored five related factors of its implementation. There is need to close the gap between highly prevalent distress unmet supportive care needs, despite frequent nurse-reported interventions to enhance resilience. The critical role of specialized education, interdisciplinary collaboration with medical team, and promotion of nurse’s own resilience will help in enhancing patient’s resilience.

## Figures and Tables

**Table 1:** Demographic data, *n* = 377.

Variables	*n*	%
Gender		
Female	365	96.8
Male	10	2.7
Age (years)		
20–29	75	19.9
30–39	146	38.7
40–49	128	34.0
50–59	27	7.2
Years of nursing experience		
More than 1 yr, less than 3 yrs.	15	4.0
More than 3 yrs, less than 5 yrs.	26	6.9
More than 5 yrs, less than 10 yrs.	76	20.2
More than 10 yrs, less than 20 yrs.	168	44.6
More than 20 yrs	91	24.1
Job title		
Deputy nursing incharge, Chief	59	15.6
Staff nurse	315	83.6
Affiliation department		
Ward nurses	130	34.5
Outpatient chemotherapy nurses	247	65.5
Education		
Junior college and vocational school	281	74.5
University	89	23.6
Graduate School	6	1.6
Qualification related to cancer nursing (Multiple answer)		
None	314	83.3
Certified Nurse (Cancer Chemotherapy Nursing)	50	13.3
Certified Nurse Specialist (Cancer nursing)	6	1.6
Certified Nurse (Palliative Care)	5	1.3
Certified Nurse (Cancer Pain Management Nursing)	2	0.5

**Table 2:** Factor patterns of nursing interventions for promoting resilience among cancer patients receiving chemotherapy.

					*n* = 377	
Factor	1	2	3	4	5	6
1st factor: Support for patients during the present situation and increasing their self-affirmation						
14. Sympathize with the patient’s positive feelings and words	0.78	0.01	0.01	−0.03	−0.04	−0.07
10. Listen to the patient describe his/her situation	0.66	0.02	−0.06	0.01	0.13	−0.07
9. Understand the personality of the patient	0.65	−0.06	0.17	−0.08	−0.02	−0.02
15. Saying positive reinforcement for patients in order to regain positive attitude	0.59	−0.04	−0.05	0.06	−0.01	0.18
11. Have a positive response to what the patient is saying or how the patient is behaving, such as ‘You made it’	0.58	−0.02	0.09	−0.02	−0.01	0.04
20. Accept a depressed state of mind by listenning to the patient’s feeling or distress	0.54	0.09	0.04	0.12	0.00	−0.07
12. Mention words or phrases that the patient would expect	0.48	−0.01	−0.04	0.01	−0.04	0.22
2nd factor: Support for self-help						
47. Provide information so that the patient can clarify his or her intention and receive treatment	−0.06	0.91	0.04	−0.05	−0.05	−0.09
48. Consider improvement measures based on the patient’s lifestyle until recovery	0.09	0.78	−0.05	−0.02	0.02	0.01
46. Intentionally create an opportunity to understand the patient’s self-determination	−0.03	0.71	0.01	−0.01	0.02	0.05
50. Understand the characteristics of patient coping behavior	0.06	0.50	0.03	0.07	0.02	0.08
49. Promote patients to self-evaluate their life in recuperation	−0.05	0.50	−0.05	0.06	0.05	0.25
3rd factor: Support for utilizing the cancer medical team						
28. Encourage patients to obtain information and consult with doctors	0.09	−0.05	0.72	0.08	0.05	−0.04
27. Consider support from other medical personal when evaluating patients	0.10	−0.05	0.72	−0.03	0.03	−0.01
29. Ask the palliative care team to cooperate in providing support for patients	−0.03	0.07	0.66	−0.05	−0.02	−0.02
30. Ask pharmacists to cooperate in providing support for patients	−0.03	−0.08	0.57	0.00	−0.01	0.23
22. Ask other professionals to intervene when patients cannot solve problems themselves	0.09	0.26	0.43	−0.07	−0.13	0.02
(psychiatric examination/treatment)						
4th factor: Support for obtaining family cooperation						
40. Ask patients whether they have physical support from their families	0.00	−0.07	−0.07	0.98	−0.06	0.08
39. Ask patients whether they have mental support from their families	0.00	0.00	−0.01	0.90	−0.01	−0.02
38. Ask family members about their feelings in supporting patients	0.05	0.20	0.13	0.43	0.09	−0.18
5th factor: Support for maintaining regular lifestyle during chemotherapy						
64. Ask the family about the patient’s lifestyle and conditions outside the hospital setting	−0.12	−0.04	0.11	0.03	0.87	−0.03
63. Ask patients about their lifestyle and conditions outside the hospital setting	0.10	−0.01	−0.10	−0.09	0.85	0.02
65. Ask about the daily lifestyle of patients and support so that their treatment can be customized based on their preferences	0.10	0.17	−0.03	0.03	0.52	0.04
6th factor: Support for interacting with cancer patients and utilizing necessary information						
2. Tell the patient that other patients also are worried by similar experiences	0.17	0.03	−0.09	−0.07	−0.12	0.61
53. Share stories related to the experience of other patients	0.08	−0.07	−0.02	0.03	0.15	0.56
31. Encourage the patient to interact with other patients and obtain information about their experiences	−0.17	0.05	0.15	0.01	0.09	0.53
34. Encourage patients to use the Cancer Consultation Support Office	−0.08	0.06	0.18	0.07	−0.07	0.46
Factor correlation	1	2	3	4	5	6
1		0.58	0.56	0.49	0.50	0.44
2			0.58	0.58	0.63	0.52
3				0.49	0.48	0.43
4					0.55	0.47
5						0.35
6						
Cronbach’s α	0.83	0.84	0.78	0.82	0.81	0.67

Factor extraction method: Maximum likelihood method, Rotation method: Promax method; (1) Mean and standard deviation is obtained dividing sum of score of each factor by number of the item.

**Table 3:** Average score, *t*-test and ANOVA for nursing interventions for promoting resilience among cancer patients receiving chemotherapy (NIPRPC).

Item		*n*	1st Factor	2nd Factor	3rd Factor	4th Factor	5th Factor	6th Factor
(Range)			(7–28)	(5–20)	(5–20)	(3–12)	(3–12)	(4–16)
Job title	Deputy nursing incharge, Chief	59	23.5 ± 2.6	15.1 ± 2.5	17.1 ± 2.5 *	8.7 ± 1.8	9.5 ± 1.8 *	11.4 ± 1.9
	Staff nurse	315	23.3 ± 2.8	14.3 ± 2.7	16.1 ± 2.4	8.4 ± 1.8	8.9 ± 1.8	11.0 ± 2.1
Have a professional qualification	yes	63	23.8	16.0 ***	16.7	8.8	9.7 **	12.2 ***
	No	314	23.2	15.6	16.2	8.4	8.9	10.8
Use of nursing journals/research papers/academic conferences for learning	Yes	230	23.8 ± 2.7	15.6 ± 2.4 **	16.7 ± 2.3	8.8 ± 1.9	9.7 ± 1.9 **	12.2 ± 1.8 **
	No	147	23.2 ± 2.8	14.2 ± 2.6	16.2 ± 2.4	8.4 ± 1.8	8.9 ± 1.8	10.8 ± 2.0

*t*-test, ***: *p* < 0.001, **: *p* < 0.01, *: *p* < 0.05.

**Table 4:** The correlation of NIPRPC between Resilience Scale for Nurses (RSN) and the number of collaborations with professionals.

Item	*n*	1st Factor	2nd Factor	3rd Factor	4th Factor	5th Factor	6th Factor
Resilience scale for nurses (total score)	368	0.325 **	0.280 **	0.323 **	0.272 **	0.197 **	0.188 **
The number of collaborations with professionals (palliative care team, psychiatry liaison)	377	0.204 **	0.213 **	0.181 **	0.170 **	0.191 **	0.184 **

Pearson’s correlation coefficient, ** *p* < 0.01, 1st factor: Support for patient to face the present situation and to increase self-affirmation; 2nd factor: Support for dealing with problems yourself; 3rd factor: Support for utilizing cancer medical team; 4th factor: Support for getting family cooperation; 5th factor: Support for maintaining ordinariness in life during chemotherapy; 6th factor: Support for interaction with cancer patients and utilization of necessary information.

**Table 5:** Multiple regression for NIPRPC.

*n* = 365												
**Predictor Variables**	**1st Factor**						**2nd Factor**					
	B	SE	β	*p*<	95% CI		B	SE	β	*p*<	95% CI	
(Variable)	15.46	1.07		0.000	13.35	17.57	8.00	1.04		0.000	5.96	10.04
Job title												
Have a professional qualification							1.12	0.35	0.16	0.001	0.44	1.81
Use of nursing journals/research papers/academic conferences for learning												
Resilience scale for nurses (total score)	0.08	0.01	0.30	0.000	0.05	0.10	0.06	0.01	0.24	0.000	0.04	0.08
The number of collaborations with professionals	0.19	0.05	0.17	0.001	0.08	0.30	0.18	0.05	0.17	0.001	0.08	0.28
Adjusted R2	0.13						0.13					
**Predictor Variables**	**3rd Factor**						**4th Factor**					
	B	SE	β	*p*<	95% CI		B	SE	β	*p*<	95% CI	
(Variable)	10.99	1.20		0.000	8.64	13.34	4.22	0.72		0.000	2.81	5.63
Job title	−0.70	0.33	−0.11	0.032	−1.34	−0.06						
Have a professional qualification												
Use of nursing journals/research papers/academic conferences for learning												
Resilience scale for nurses (total score)	0.07	0.01	0.30	0.000	0.05	0.09	0.04	0.01	0.26	0.000	0.03	0.06
The number of collaborations with professionals	0.13	0.05	0.13	0.007	0.04	0.22	0.09	0.04	0.13	0.010	0.02	0.17
Adjusted R2	0.13						0.09					
**Predictor Variables**	**5th Factor**						**6th Factor**					
	B	SE	β	*p*<	95% CI		B	SE	β	*p*<	95% CI	
(Variable)	5.98	0.74		0.000	4.54	7.43	7.91	0.83		0.000	6.27	9.54
Job title												
Have a professional qualification							0.77	0.34	0.14	0.023	0.10	1.44
Use of nursing journals/research papers/academic conferences for learning	0.54	0.20	0.14	0.007	0.15	0.93	0.60	0.28	0.14	0.032	0.05	1.15
Resilience scale for nurses (total score)	0.03	0.01	0.15	0.004	0.01	0.04	0.02	0.01	0.12	0.016	0.00	0.04
The number of collaborations with professionals	0.10	0.04	0.13	0.011	0.02	0.17	0.10	0.04	0.12	0.015	0.02	0.19
Adjusted R2	0.08						0.11					

Note: Job title (staff nurse: 0, Deputy nursing incharge, Chief: 1), Have a professional qualification (no: 0, yes: 1) Use of nursing journals/research papers/academic conferences for learning (no: 0, yes: 1), B: Partial regression coefficient, SE: Staandard Error, CI: Confidence Interval. 1st factor: Support for patient to face the present situation and to increase self-affirmation; 2nd factor: Support for dealing with problems yourself; 3rd factor: Support for utilizing cancer medical team; 4th factor: Support for getting family cooperation; 5th factor: Support for maintaining ordinariness in life during chemotherapy; 6th factor: Support for interaction with cancer patients and utilization of necessary information.

## Data Availability

The data presented in this study are available on request from the corresponding author. The data are not publicly available due to privacy.
